# Sequencing and analysis of the complete mitochondrial genome of *Rana amurensis* (Anura: Ranidae)

**DOI:** 10.1080/23802359.2017.1357444

**Published:** 2017-07-25

**Authors:** Peng Liu, Heng Wang, Wenge Zhao

**Affiliations:** College of Life Science and Technology, Harbin Normal University, Harbin, Heilongjiang, P.R. China

**Keywords:** Ranidae, mitogenome, *Rana amurensis*, phylogenetic tree

## Abstract

In this study, the complete mitogenome sequence of *Rana amurensis* (Anura: Ranidae) is determined using long PCR. It is a circular molecule of 20,571 bp in length (GenBank accession no. MF370348). The complete mtDNA sequence of *R. amurensis* contained 2 rRNA genes (12S rRNA and 16S rRNA), 22 tRNA genes, 13 protein-coding genes (PCGs) and 2 control regions (D-loops). The nucleotide composition was 28.6% A, 26.8% C, 14.0% G and 30.6% T. Mitochondrial genome analyses based on NJ method yield phylogenetic trees, including that 20 reported family Ranidae frogs. These molecular data presented here provide a useful tool for systematic analyses of genus *Rana*.

Heilongjiang brown frog (*Rana amurensis*) is mainly found in northern Asia. It ranges across western Siberia, as well as north-eastern China, north-eastern Mongolia, Korean Peninsula and Sakhalin (Zhao et al. 2008; Fei et al. 2012). The resources of *R. amurensis* have been declining recently because of excessive capturing considering its high economic value in China (Zhao et al. 2008). Mitochondrial 12S rRNA, 16S rRNA and cytochrome b gene of *R. amurensis* have been amplified and sequenced to reveal the population differentiation and phylogenetic relationships with other frogs (Tanaka-Ueno et al. 1998; Yang et al. 2001; Sumida et al. 2003; Che et al. 2007).

In this paper, the complete mitochondrial genome of *R. amurensis* is sequenced with a muscle sample using a primer walking strategy and the long and accurate PCR. The specimen was collected from Heilongjiang Province of China (45°17'16.06''N, 127°33'09.81''E) and was stored in Zoological and Botanical Specimen Museum of Harbin Normal University (its accession number is HRB1504006).

The complete mtDNA of *R. amurensis* is 20,571 bp in length and contains 2 rRNA genes (12S rRNA and 16S rRNA), 22 tRNA genes, 13 protein-coding genes (PCGs) and 2 control regions (D-loops). The nucleotide composition is 28.6% A, 26.8% C, 14.0% G and 30.6% T. The accurate annotated mitochondrial genome sequence was submitted to GenBank with accession number MF370348.

Within the mitochondrial genome of *R. amurensis*, there are five reading frame overlaps (share 1–9 nucleotides) and seven intergenic spacers (range from 1 to 190 bp). Except for *ND6* and 8 tRNA genes, all other mitochondrial genes were encoded on the heavy strand (H strand). In 13 protein-coding genes, except *ND1* begins with ATA, *ND2* begins with ATT, *COI* and *ND4L* begin with GTG, and the other nine genes begin with ATG as start codon. *ND1*, *COII*, *ATP6*, *COIII*, *ND3* and *ND4* end with a single stop nucleotide T; *ND2* ends with TAG; *COI* and *ND6* ends with AGG; *ATP8*, *ND4L* and *CYTB* ends with TAA; and *ND5* ends with AGA. The 22 tRNA genes with the size ranging from 65 to 73 bp are interspersed along the whole genome. The sequence length of the 12S and 16S rRNA is 929 and 1577 bp, and D-loop regions are 2324 and 2695 bp. In the WANCY cluster of tRNA genes, a 28 bp sequence is considered as the putative L strand replication origin (OL).

Mitochondrial genome analyses based on MP, ML and NJ yielded identical phylogenetic trees, including that 20 reported family Ranidae frogs (Figure 1). It appeared that *R. amurensis* and *R. kunyuensis* formed a monophyletic group and they have a close genetic relationship. But *R. huanrensis* is formed a group with *R. asiatica*, *R. cf chensinensis*, *R. kukunoris* and *R. dybowskii*. This study will facilitate the further research on comparing the genetic structure of *R. amurensis* and systematic analyses with *R. coreana*, previously included in this species as a subspecies (Song et al. 2006), and we also wish that our study contributes to the protection of *R. amurensis*.

**Figure 1.**
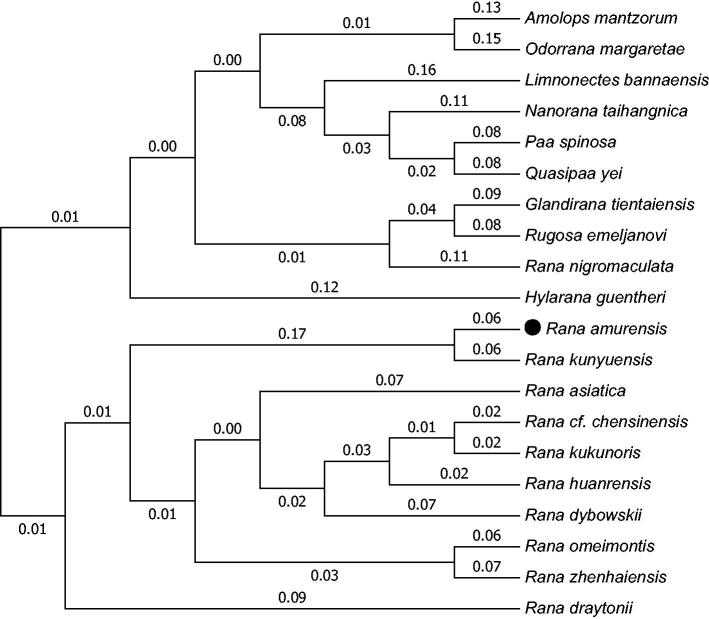
Phylogenetic tree generated using the neighbour joining method based on complete mitochondrial genomes of some species in Anura: Ranidae. *Amolops mantzorum* (NC_024180), *Odorrana margaretae* (NC_024603), *Limnonectes bannaensis* (NC_012837), *Nanorana taihangnica* (NC_024272), *Paa spinosa* (FJ432700), *Quasipaa yei* (NC_024843), *Glandirana tientaiensis* (NC_025226), *Rugosa emeljanovi* (KU641020), *Rana nigromaculata* (NC_002805), *Hylarana guentheri* (NC_024748), *R. amurensis* (MF370348), *Rana kunyuensis* (NC_024548), *Rana asiatica* (NC_032328), *Rana cf chensinensis* (NC_023529), *Rana kukunoris* (NC_032330), *Rana huanrensis* (NC_028521), *Rana dybowskii* (NC_023528), *Rana omeimontis* (NC_032329), *Rana zhenhaiensis* (NC_032332) and *Rana draytonii* (NC_028296).
